# Expression of a *NGATHA1* Gene from *Medicago* *truncatula* Delays Flowering Time and Enhances Stress Tolerance

**DOI:** 10.3390/ijms21072384

**Published:** 2020-03-30

**Authors:** Tao Guo, Shumin Wang, Yinruizhi Li, Jianbo Yuan, Lixin Xu, Tiejun Zhang, Yuehui Chao, Liebao Han

**Affiliations:** 1 College of Grassland Science, Beijing Forestry University, Beijing 100083, China; gunter2014@bjfu.edu.cn (T.G.); liyinruizhi@163.com (Y.L.); yuanjingan113@163.com (J.Y.); lixinxu@bjfu.edu.cn (L.X.); zhangtiejun@bjfu.edu.cn (T.Z.); 2 College of Agro-Grassland Science, Nanjing Agricultural University, Nanjing 210095, China; 2016220001@njau.edu.cn

**Keywords:** NGA1, *M. truncatula*, late flowering, branch, abiotic stress response

## Abstract

Shoot branching is one of the most variable determinants of crop yield, and the signaling pathways of plant branches have become a hot research topic. As an important transcription factor in the B3 family, NGATHA1 (NGA1), plays an important role in regulating plant lateral organ development and hormone synthesis and transport, but few studies of the role of this gene in the regulation of plant growth and stress tolerance have been reported. In this study, the *NGA1* gene was isolated from *Medicago truncatula (Mt)* and its function was characterized. The cis-acting elements upstream of the 5′ end of *MtNGA1* and the expression pattern of *MtNGA1* were analyzed, and the results indicated that the gene may act as a regulator of stress resistance. A plant expression vector was constructed and transgenic Arabidopsis plants were obtained. Transgenic Arabidopsis showed delayed flowering time and reduced branching phenotypes. Genes involved in the regulation of branching and flowering were differentially expressed in transgenic plants compared with wild-type plants. Furthermore, transgenic plants demonstrated strong tolerances to salt- and mannitol-induced stresses, which may be due to the upregulated expression of *NCED3* (*NINE-CIS-EPOXYCAROTENOID DIOXYGENASE 3*) by the *MtNGA1* gene. These results provide useful information for the exploration and genetic modification use of *MtNGA1* in the future.

## 1. Introduction

The B3 superfamily consists of plant-specific transcription factors that are also present in green algae, mosses [1], liverworts, ferns and gymnosperms, all of which are vascular plants. Compared to families with animal and bacterial orthologs, much less is known about the plant-specific transcription factor families. For example, the MYB, Basic helix-loop-helix (bHLH) and MADS families of transcription factors are present in plants as well as animals and yeast [2]. The B3 family encompasses four characterized families, the auxin response factor (ARF), leafy cotyledon 2–abscisic acid insensitive 3–val (LAV), related to ABSCISIC ACID INSENSITIVE3 (ABI3) and VIVIPAROUS1 (VP1) (RAV) and reproductive meristem (REM) families [3]. The B3 family plays a central role in plant life, and its members are characterized by the presence of B3 DNA-binding domains (DBDs) [4]. This domain was initially named because it was the third basic domain to be discovered in the maize gene *VP1* (*VIVIPAROUS1*) [5]. The B3 domain of VP1 carries out sequence-specific DNA binding activity [6].

The B3 family has been investigated for its role in regulating pathways such as flower organ and leaf development, meristem division and differentiation, and the stress response. The ARF has a demonstrated a role in senescence and floral organ abscission: In Arabidopsis, *AtARF1*, *AtARF7* and *AtARF19* are induced by senescence. *arf1* and *arf2* mutant plants exhibited delays in several processes, plant aging, the initiation of flowering, rosette leaf senescence, floral organ abscission and silique ripening; *arf1* mutation increased transcription of *AUXIN/INDOLE-3-ACETIC ACID (Aux/IAA)* genes in *Arabidopsis* flowers [7]. The *Arabidopsis LEAFY COTYLEDON2* (*LEC2*) gene, belonging to the LAV family, is a central transcriptional regulator of both early and late embryo development, providing a cellular environment for embryo development, and is also required for the maintenance of suspensor morphology and specification of cotyledon identity [8]. In Arabidopsis, three members of the LAV family, LEC2, FUSCA (FUSCA3), and ABSCISIC ACID INSENSITIVE 3 (ABI3), are involved in zygotic and somatic embryogenesis. Meanwhile, the expression of *MtLEC2*, *MtFUSCA3*, and *MtABI3* in *M. truncatula* is induced during somatic embryogenesis, and expression of *MtFUSCA3* and *MtABI3* occurs precisely at 10 days after pollination during seed development [9]. *CaRAV1*, *ZmRAV1*and *SlRAV2* have been confirmed to be involved in pathways against stress, such as high salt, drought, low temperature or infection [10,11,12]. AtREM1 plays a role in the organization of reproductive meristems and during flower organ development, being preferentially expressed in inflorescence apices [13].

The RAV family of the B3 transcription factor superfamily includes the RAV, TEMPRANILLO (TEM) and NGATHA (NGA) subfamily [3]. NGA1 specifically binds the 5′-CACCTG-3′ DNA sequence [4], and NGA family have been shown to be involved in the development of flower organs and leaves, cell proliferation in lateral organs, and seed development. BrNGA1 has been suggested to be involved in negatively regulating cell proliferation during lateral organ and root growth, and *BrNGA1* transgenic seedlings displayed de-etiolation phenotypes under dark conditions [14]. NGAs and CINCINNATA-class-thermoresponsive cationic copolymers (CIN-TCPs) are co-expressed at many stages of leaf development, and their absence causes persistent marginal growth in leaves, cotyledons and floral organs [15]. Plants overexpressing individual Arabidopsis *NGATHA* genes (*AtNGA1* to *AtNGA4*) displayed small, narrow lateral organs, and decreased cell numbers in the lateral organs in contrast to an *nga1, nga2, nga3, nga4* quadruple mutant [16]. Arabidopsis plants overexpressing *BrNGA1* also displayed markedly reduced lateral organ and root growth compared with growth in wild-type plants [17]. The *NGA* genes are involved in stigma development and may also may act as a carpel identity factors [18,19]. As NGA1 promotes a general differentiation program, the *nga* quadruple mutant can be interpreted as less differentiated and more meristematic than the wild-type plant; *nga* quadruple mutant petals were developmentally delayed, the mutant leaves were serrated, the leaf cell cycle was longer for the mutant plants and a lower cell division rate was observed in *NGAox* leaves [19]. In addition, NGA impacts auxin transport through altering the activity of protein kinases that regulate the cellular localization of auxin efflux regulators [20]. The response of NGA to the auxin-based signaling pathway in turn affects the activation of the style program in the apical gynoecium domain [21,22]. Overexpression of *NGATHA-like* (*NGAL1*) led to transgenic plants with a conspicuous defective flower phenotype of petal loss [23]. Overexpression of *NGAL2* significantly decreased seed size compared to that of the wild-type plants by restricting cell proliferation in the integuments of ovules and developing seeds, while *NGAL3* overexpression increased size, although *NGAL2* and *NGAL3* act in the same pathway with the seed size regulator *KLUH (KLU)* to regulate seed growth [24]. In Arabidopsis, NGA1 activates the *NCED3* gene by an NGA-binding element (NBE) in the 5′ untranslated region (5′ UTR) of the *NCED3* promoter, and then positively regulates abscisic acid (ABA) accumulation during dehydration stress due to the *NCED3* gene, which encodes a key ABA biosynthetic enzyme [25].

In summary, NGA proteins affect plant organ development and regulate hormone synthesis and transport and drought-stress tolerance pathways. Many of the functions of NGA proteins have been discovered in recent years; these functions include prediction functions in many pathways, which suggests a strong exploration potential. Therefore, research on NGA in the model plant *M. truncatula* is meaningful, especially because the involvement of MtNGA1 in the regulatory network of flowering time and branching development, which is affected by strigolactone, has not been reported. Additionally, we found the function of MtNGA1 in the response to abiotic stress. This study can broaden the functional exploration of NGA proteins in different species, laying a foundation to improve the utilization of genetic resources and the study of high-quality forage alfalfa worldwide.

## 2. Results

### 2.1. Identification and Homology Analysis of the MtNGA1 Gene in M. truncatula

**Figure 1:**
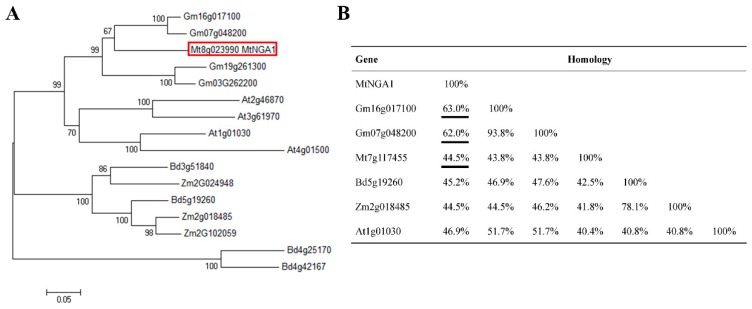
Phylogenetic tree analysis and homologous relation between NGATHA1 (NGA1) proteins from different species. (**A**) Phylogenetic tree analysis of 16 NGA1 proteins. Sequences were obtained from the Plant Transcription Factor Database (PlantTFDB). The tree was constructed using the neighbor-joining method with MEGA6.0 software. (**B**) Sequence homology of NGA1 proteins in the indicated species. MtNGA1 in *M. truncatula* was boxed. Gene accession numbers: *MtNGA1* (Mt8g023990); *Arabidopsis thaliana*(At): At2g46870, At3g61970, At1g01030, At4g01500; *Medicago truncatula* (Mt): Mt7g117455; *Glycinemax* (Gm): Gm16g017100, Gm07g048200, Gm19g261300, Gm03g262200; Zea mays (Zm): Zm2g024948, Zm2g018485, Zm2g102059; *Brachypodium distachyon* (Bd): Bd3g51840, Bd5g19260, Bd4g25170, Bd4g42167.

According to the National Center for Biotechnology Information (NCBI)database and Plant Transcription Factor Database (PlantTFDB), the DNA fragment containing *MtNGA1* Coding Sequences (CDS) was cloned and ligated into a cloning vector to construct the recombinant plasmid pEASY-MtNGA1. We analyzed the structure of MtNGA1, which contains only one B3 domain, and belongs to the RAV subfamily of the B3 transcription factor family. Using MEGA6.0 software, the phylogenetic tree of the NGA1 sequences was constructed to evaluate the phylogenetic relationships among soybean NGAs, Arabidopsis NGAs, maize NGAs, *M. truncatula* NGA and *Brachypodium distachyon* NGAs with a neighbor-joining method, and the results showed that the MtNGA1 protein has the highest homology (63%) with Gm16g017100 from soybean (Figure 1A,B). Among the B3 families in *M. truncatula*, Mt7g117455 had the highest homology with MtNGA1 (only 44.5%) (Figure 1B). This result may indicate that MtNGA1 plays a role distinct from other B3 family transcription factors from *M. truncatula.*

### 2.2. Subcellular Localization of MtNGA1

**Figure 2:**
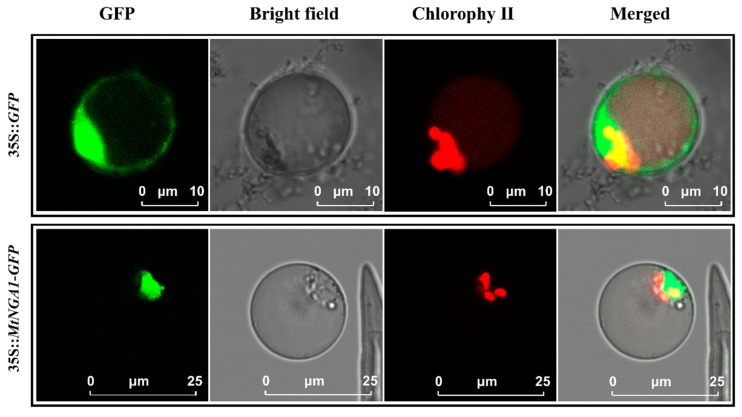
Subcellular localization of the MtNGA1 proteins. Under the green fluorescence excitation channel, MtNGA1-GFP obviously localized to the nucleus (green). 35S::*GFP* scale bars = 10 μm, 35S::*MtNGA1-GFP* scale bars = 25 μm.

PlantTFDB and UniProt predicted that MtNGA1 is a transcription factor in the nucleus. An MtNGA1-GFP fusion protein was expressed in *M. truncatula* protoplast cells as described by Yoo [26]. The GFP signal in the protoplasts was detected by confocal laser scanning microscopy. The results showed that a strong GFP signal was only detected from the nucleus when the MtNGA1-GFP fusion protein was overexpressed, while the GFP signal was found throughout the whole cell in cells expressing the GFP protein (Figure 2). These results suggested that MtNGA1 is a nuclear protein, which is consistent with the predicted results.

### 2.3. Promoter Analysis of MtNGA1

**Figure 3:**
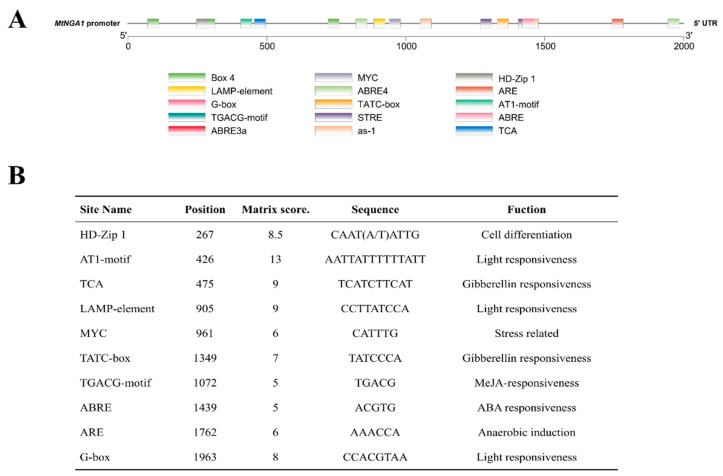
Promoter analysis of *MtNGA1* gene (**A**) Cis acting elements contained in *MtNGA1* gene promoterand. (**B**) Prediction of binding sites for the transcription factors in the *MtNGA1* gene promoter.

To analyze the potential function and regulatory pathway of MtNGA1, we obtained the 5′ upstream sequence (2000 bp in length) of the *MtNGA1* gene from NCBI and then identified cis-acting elements in the region with PlantCARE. The results showed that the most abundant binding sites were present in the region involved in stress resistance and light responses (Figure 3), which indicated that this gene may act as a regulator in stress.

### 2.4. Expression Analysis of MtNGA1

**Figure 4:**
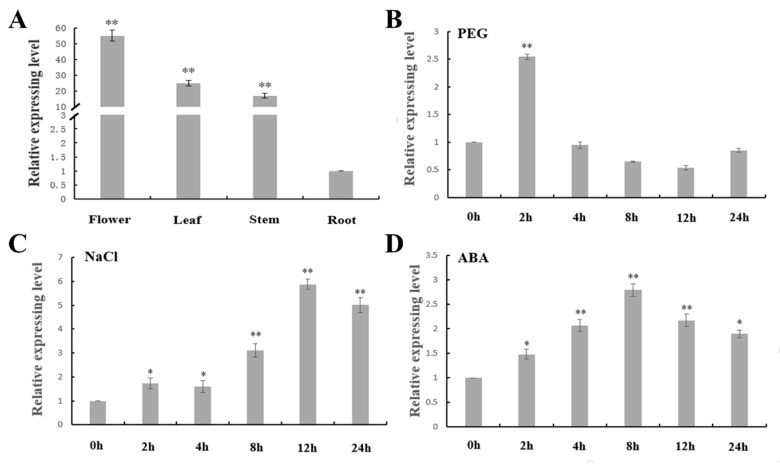
Expression profiles of *MtNGA1* in *M. truncatula*. (**A**) Relative expression levels of *MtNGA1* in different plant organs: the flowers, leaves, stems, and roots. (**B**) Relative expression levels of *MtNGA1* under 15% Polyethylene Glycol (PEG) treatments. (**C**) Relative expression levels of *MtNGA1* under 200 mM NaCl treatment. (**D**) Relative expression levels of *MtNGA1* under 100 µM ABA treatment. All samples were harvested at 0 h, 2 h, 4 h, 8 h, 12 h, and 24 h. The values are the means ± standard deviations (SDs; *n* = 3). * indicates significant differences of the means at *p* < 0.05 between flower, leaf or stem expression and root expression determined by expression analysis (*n* = 3). ** indicates significant differences of the means at *p* < 0.01 between each treated sample and untreated sample determined by expression analysis (*n* = 3).

To identify the expression pattern of *MtNGA1* in different tissues, different tissue samples were harvested from *M. truncatula* at the flowering stage. Based on the results of cis-acting element prediction analysis, *M. truncatula* samples under different abiotic stresses and ABA treatment conditions were also harvested. qRT-PCR was performed, and the results showed that *MtNGA1* was differentially expressed in different tissues. Its gene expression was highest in flowers and lowest in roots (Figure 4A). *MtNGA1* showed increased expression levels under two abiotic stresses: Polyethylene Glycol (PEG) and NaCl. Under PEG conditions, the expression of *MtNGA1* rapidly increased, reaching a peak at 2 h, and then decreased to normal levels (Figure 4B), indicating that MtNGA1 plays a regulatory role in the early stage of PEG-induced stress. For NaCl treatments, expression of the *MtNGA1* gene continued and reached its peak at 12 h. The expression level of *MtNGA1* was lower at 24 h than at 12 h but was still much higher than that without treatment (Figure 4C). These results implied that MtNGA1 is involved in regulation and the response to salt stress. With exogenous ABA treatment, *MtNGA1* expression levels were enhanced (highest at 8 h). These results suggest that MtNGA1 may play a role in regulating the response to abiotic stresses and ABA (Figure 4D).

### 2.5. Overexpression of MtNGA1 Delayed Flowering Time and Reduced the Number of Arabidopsis Branches

**Figure 5:**
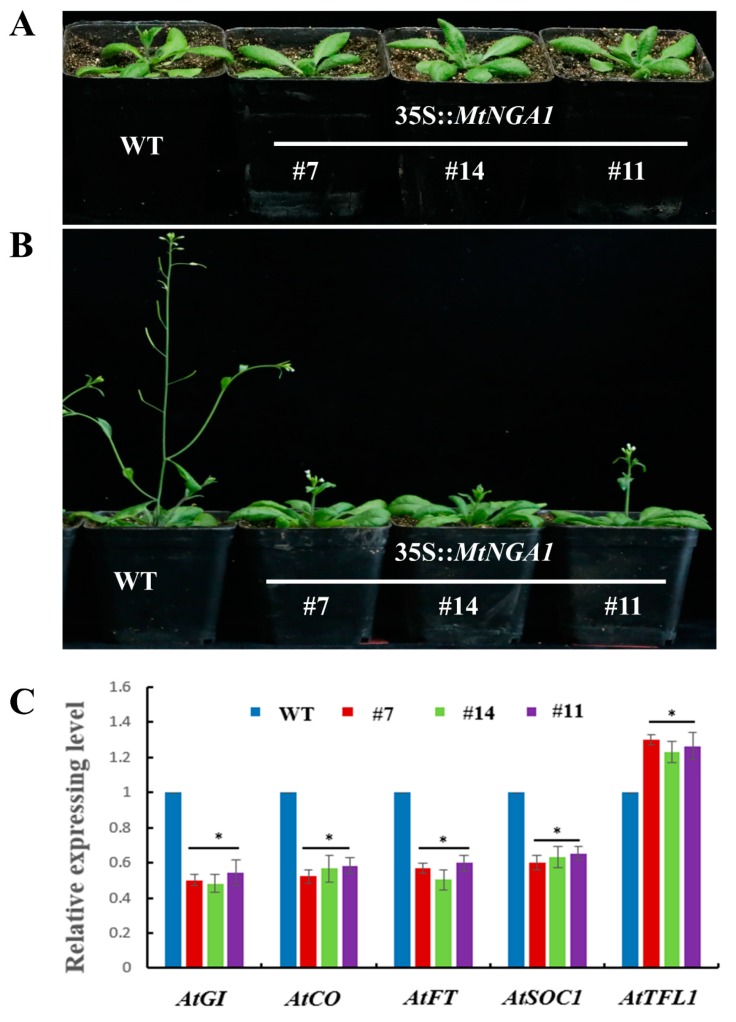
Comparative analysis of flowering time between transgenic and wild-type Arabidopsis. (**A**) Phenotype of transgenic Arabidopsis expressing the *MtNGA1* gene and wild-type Arabidopsis at 30 days. (**B**) Comparison of the phenotypes of transgenic Arabidopsis and wild-type Arabidopsis at 40 days. (**C**) Expression analysis of genes related to flowering in transgenic and wild-type (WT) plants by qRT-PCR. *GI, GIGANTEA. CO, CONSTANS. FT, Flowering locus T. SOC1, SUPPRESSOR OF OVEREXPRESSION OF CONSTANS 1. TFL1, TERMINAL FLOWER 1*. The values are the means ± SD (*n* = 3). * indicates significant differences of the means at *p* < 0.05 between each level in transgenic lines and WT plants determined by expression analysis (*n* = 3).

**Figure 6:**
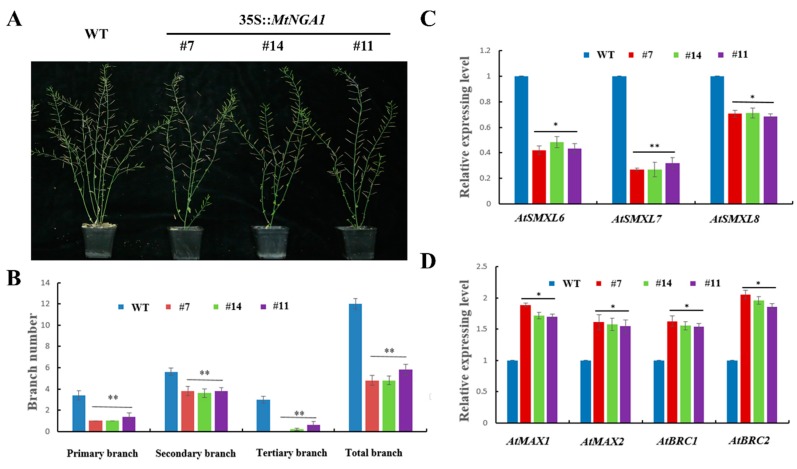
Branching related phenotype and gene expression analysis of in transgenic and WT Arabidopsis. (**A**) Branching phenotypes of WT and transgenic Arabidopsis. (**B**) The numbers of branches in WT and transgenic plants. The values are the means ± SD (*n* = 30). ** indicates significant differences of the means at *p* < 0.01 between branch number in transgenic lines and WT plants (*n* = 30). (**C**) Expression analysis of *AtSMAX1-LIKE6 (SMXL6)*, *AtSMXL7* and *AtSMXL8* in transgenic and WT Arabidopsis. (**D**) Expression analysis of *AtMORE AXILLARY GROWTH 1 (MAX1)*, *AtMAX2*, *AtBRANCHED 1 (BRC1)* and *AtBRC2* in transgenic and WT Arabidopsis. The values are the means ± SDs (*n* = 3). * indicates significant differences of the means at *p* < 0.05 between the expression level of each gene in transgenic lines and WT plants (*n* = 3). ** indicates significant differences in the means at *p* < 0.01 between the expression level of each gene in transgenic lines and WT plants (*n* = 3).

We selected three transgenic lines with the highest expression of the target gene (MtNGA1-7, MtNGA1-14 and MtNGA1-11) as the experimental subjects. *MtNGA1*-transgenic Arabidopsis showed late flowering and reduced branch number phenotypes (Figure 5 and Figure 6). The flowering time of the transgenic plants was 8–10 days later than that of the wild-type (Figure 5A,B). Meanwhile, the transgenic lines showed a lower bolting rate than the wild-type plants. To detect other genes involved in flowering control in transgenic Arabidopsis, *GIGANTEA (GI)*, *CONSTANS (CO), Flowering locus T (FT)*, *SUPPRESSOR OF OVEREXPRESSION OF CONSTANS 1 (SOC1)* and *TERMINAL FLOWER 1 (TFL1)* were analyzed by qRT-PCR. The expression levels of *GI*, *CO*, *FT* and *SOC1* were downregulated, while those of *TFL1* was upregulated (Figure 5C). These results are consistent with the late flowering phenotype of the transgenic plants and indicate that MtNGA1 is involved in the regulation of flowering time.

To analyze branch number, the primary, secondary and tertiary branches in plants of these lines were counted (Figure 6A,B). Under normal growth conditions, the number of primary and secondary branches in the transgenic plants was reduced by 2–3 compared with that in the WT plants, and the primary branch numbers of the MtNGA1-7 and MtNGA1-14 lines were only one. For the MtNGA1-11 lines, a minority of plants produced another weak branch from the basal rosette, but could not produce seeds. The MtNGA1-7 lines could not produce tertiary branches, while the MtNGA1-14 and MtNGA1-11 lines had 1–2 small tertiary branches, and WT plants had 3–4 tertiary branches (Figure 6B). The total branch numbers in transgenic lines were obviously also significantly lower than those in the WT plants (Figure 6B).

We also analyzed expression of the following genes involved in branching regulation and development in transgenic Arabidopsis: *AtSMAX1-LIKE6 (SMXL6)*, *AtSMXL7*, *AtSMXL8*, *AtMORE AXILLARY GROWTH 1 (MAX1)*, *AtMAX2*, *AtBRANCHED 1 (BRC1)* and *AtBRC2*. In Arabidopsis, *AtSMXL6*, *AtSMXL7*, and *AtSMXL8* positively regulate branch numbers, and the expression levels of *AtSMXL6*, *AtSMXL7* and *AtSMXL8* in the transgenic plants were significantly decreased (Figure 6C). *MAX* and *BRC* can inhibit branching, and the qRT-PCR results showed that *AtMAX1* and *AtMAX2* as well as *AtBRC1* and *AtBRC2* were upregulated in the transgenic plants (Figure 6D). These results are consistent with the reduced branch number phenotype in the transgenic plants. Furthermore, these results showed that MtNGA1 is involved in regulating branch number by the negative regulation of *AtSMXL6*, *AtSMXL7* and *AtSMXL8*, and the positive regulation of *AtMAX1*, *AtMAX2*, *AtBRC1* and *AtBRC2*.

### 2.6. MtNGA1 Enhanced Resistance to Mannitol-Induced and NaCl Stresses

**Figure 7:**
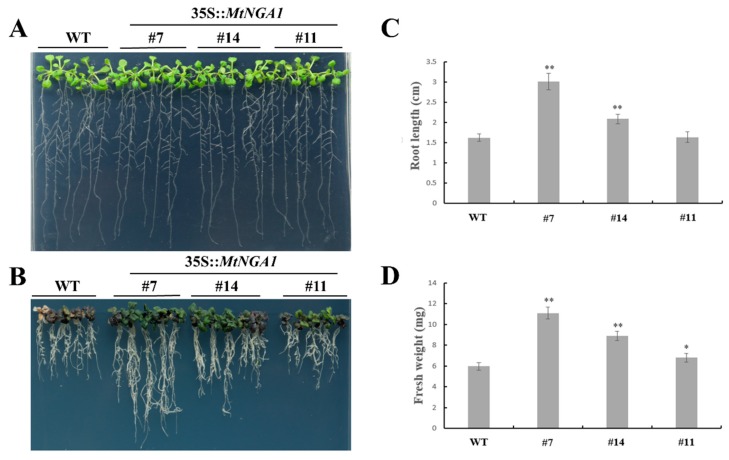
Phenotypic comparison of wild-type and transgenic plants under 500 mM mannitol treatment. (**A**) Five-day-old seedlings of three transgenic lines and WT plants were planted on 1/2 MS medium without mannitol and grown for 7 days. (**B**) Five-day-old seedlings of three transgenic lines and WT plants were planted on 1/2 MS medium with 500 mM mannitol and grown for 7 days. (**C**) The root lengths of plants in the transgenic lines and WT plants were measured 7 days after 500 mM mannitol treatment. (**D**) The fresh weights of plants in the transgenic lines and WT plants were measured 7 days after 500 mM mannitol treatment. The values are means ± SD (*n* = 30). * indicates significant differences of the means at *p* < 0.05 between transgenic lines and WT plants (*n* = 30). ** indicates significant differences of the means at *p* < 0.01 between transgenic lines and WT plants (*n* = 30).

Based on the expression patterns of *MtNGA1*, tolerance to mannitol and NaCl was estimated in WT and transgenic Arabidopsis. On 1/2 MS medium, all transgenic lines and WT plants showed similar phenotypes (Figure 7A). When mannitol at a final concentration of 500 mM was added to the 1/2 MS medium, the wild-type seedlings gradually turned purple and yellow, and finally died, and their roots were shorter than those from the majority of the transgenic lines (Figure 7B). The average root lengths of the MtNGA1-7 and MtNGA1-14 transgenic lines were 85% and 28% longer than that of WT plants, respectively, while the differences in root length between the MtNGA1-11 transgenic lines and wild-type plants ware not significant (Figure 7C). Compared with that of the WT plants, the fresh weights of the MtNGA1-7, MtNGA1-14 and MtNGA1-11 transgenic lines were 85%, 48% and 14% higher, respectively (Figure 7D). In general, *MtNGA1* transgenic plants exhibited enhanced tolerance to mannitol-induced stress compared to wild-type plants.

**Figure 8:**
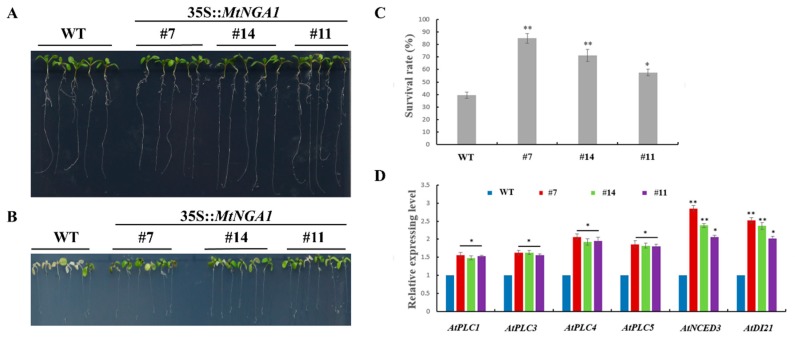
Responses of WT and transgenic plants to NaCl treatment. (**A**) Five-day-old seedlings of transgenic lines and WT plants were planted on 1/2 MS medium without NaCl and grown for 3 days. (**B**) Five-day-old seedlings of transgenic lines and WT plants were planted on 1/2 MS medium with 200 mM NaCl and grown for 3 days. (**C**) The survival rates of transgenic lines and WT plants were measured 3 days after 200 mM NaCl treatment (*n* = 30). (**D**) Expression analysis of *ATPLC1*, *ATPLC3*, *ATPLC4*, *ATPLC5*, *AtNCED3* and *AtDI21* in the transgenic lines and WT plants (*n* = 3), *PLC*, *phosphatidylinositol-specific phospholipase C*. *NCED3, NINE-CIS-EPOXYCAROTENOID DIOXYGENASE 3. DI21, drought-induced 21*; The values are the means ± SDs. * indicates significant differences of the means at *p* < 0.05 between transgenic lines and WT plants. ** indicates significant differences of the means at *p* < 0.01 between transgenic lines and WT plants.

For salt treatment, five-day-old seedlings were cultured on 1/2 MS medium containing 200 mM NaCl for three days. The leaves of wild-type plants gradually turned white or died, but most of the leaves of plants in transgenic lines remained green (Figure 8B), which indicated that the transgenic lines were less sensitive to NaCl stress. The survival rates of the MtNGA1-7, MtNGA1-14 and MtNGA1-11 transgenic lines were 84%, 71% and 57%, respectively, but the survival rate in WT plants was only 39% (Figure 8C). These results indicate that the MtNGA1 protein may be involved in the response to high salt stress, and that overexpression of the *MtNGA1* gene is helpful to improve the tolerance of plants to salt stress.

To further explore the potential mechanism by which MtNGA1 regulates tolerance to mannitol and salt stresses, we used qRT-PCR to detect changes in the expression of *AtPhosphatidylinositol-specific phospholipase C (PLC1)*, *AtPLC3*, *AtPLC4*, *AtPLC5*, *AtNCED3* and *AtDrought-induced 21 (DI21)*, which are related to abiotic stresses. All six genes selected were upregulated in the transgenic lines compared with the WT plants under normal conditions (Figure 8D). These results showed that the overexpression of the *MtNGA1* gene directly or indirectly induced the expression of *AtPLC1*, *AtPLC3*, *AtPLC4*, *AtPLC5*, *AtNCED3* and *AtDI21* in the transgenic plants, which affected the tolerances of the plants to abiotic stresses.

### 2.7. MtNGA1 Reduced the Sensitivity to Exogenous ABA

**Figure 9:**
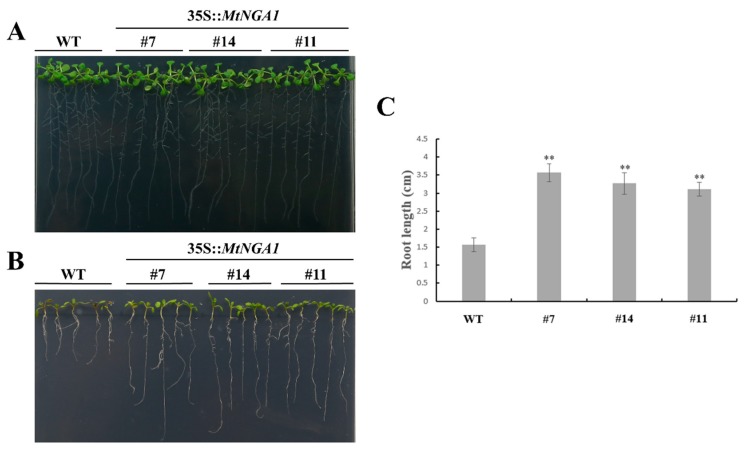
Phenotype assay of transgenic seedlings treated with ABA. (**A**) Five-day-old seedlings of transgenic lines and WT plants were planted on 1/2 MS medium without ABA and grown for 12 days. (**B**) Five-day-old seedlings of transgenic lines and WT plants were planted on 1/2 MS medium with 20 μM ABA and grown for 12 days. (**C**) The primary root of transgenic lines and WT plants were measured at 12 days after 20 μM ABA treatment. The values are the means ± SDs. * indicates significant differences of the means at *p* < 0.05 between transgenic lines and WT plants (*n* = 30). ** indicates significant differences of the means at *p* < 0.01 between transgenic lines and WT plants (*n* = 30).

Notably, we noticed that the *MtNGA1* gene exhibited increased expression with ABA treatment. To analyze whether the enhanced tolerance of the transgenic plants to abiotic stresses depends on the ABA pathway, we next studied the phenotypes of transgenic plants treated with exogenous ABA. The results revealed that each plant showed a similar phenotype on ½ MS medium. In the presence of exogenous ABA, the growth of the WT and transgenic plants was significantly inhibited, but the negative effects on the WT plants were more pronounced than those on the *MtNGA1* transgenic lines (Figure 9A,B). The three transgenic lines were less sensitive to ABA than the WT plants, and the roots of transgenic plants were much longer than those of the WT plants (Figure 9C). As a key gene in the control of ABA biosynthesis, *AtNCED3* showed increased expression in the transgenic plants (Figure 8D). Taken together, these results suggest that the enhanced tolerance in transgenic plants is at least partly dependent on reduced sensitivity to ABA.

## 3. Discussion

Analysis of the *M. truncatula* genome found 113 B3 genes in the chromosome of *M. truncatula*. The transcription factor NGA1 belongs to the RAV subfamily of the B3 superfamily, but contains only one B3 domain, unlike other RAV proteins which contain both APETALA2 (AP2) and B3 domains [21]. As the model plant of the legume family, MtNGA1 has high homology with Gm16g017100 and Gm07g048200, as shown through an evolutionary tree and homology analysis of NGA proteins among different species. Therefore, the study of MtNGA1 may lay a foundation for the functional exploration of the soybean NGA family.

In a subcellular localization assay, green fluorescence from the pSAT6::MtNGA1-GFP fusion protein was observed in the nucleus, which is consistent with previous predictions and the location of the Arabidopsis *NGA* gene [23], as it functions as a transcription factors.

Before exploring the functions of MtNGA1, we first analyzed its promoter sequence. The promoter sequence of *MtNGA1* was predicted to mainly include the following cis-element sequences: Homeodomain-leucine zipper (HD-Zip1), the AT1-motif, TCA, the LAMP element, MYC, the TATC box, the TGACG motif, ABA-responsive element (ABRE), Auxin responsive element (ARE), and G-box. The HD-Zip1 element is involved in the differentiation of palisade mesophyll cells. The AT1 motif and LAMP element are photosynthetic response elements. TCA is involved in salicylic acid responsiveness. MYC elements, which lie in the promoter of *C-repeat binding factor (CBF3)*, specifically the recognition sequences of inducer of CBF expression 1 (ICE1), are involved in cold regulation signaling pathways and regulate plant resistance ability to cold stress [27]. The TATC box cis-acting element is involved in gibberellin responsiveness. The TGACG motif is involved in Methyl Jasmonate (MeJA) responsiveness. The ABRE element is involved in plant responses to drought and ABA [28].The ARE cis-regulatory elements are auxin response elements, which are essential for anaerobic induction. The G-Box is involved in light responsiveness [29]. In conclusion, the expression of the transcription factor MtNGA1 may be involved in regulating plant growth, development and interaction with the environment.

Among different organs of the *M. truncatula* plant, *MtNGA1* expression was highest in flowers. *NGAL1* was expressed mainly in the filament of the stamen in flower tissues [23,30]. *AtNGA*, *EcNGA* (*Eschscholzia californica*) and *NbNGA* (*Nicotiana benthamiana*) showed parallel expression patterns, and could be detected in developing flowers, developing ovules and the growing apical gynoecium, with a small degree of expression at the distal end of the petals [30]. *BrNGA1* transgenic plants also displayed smaller and distinctively narrower flowers than the wild-type plants [17]. The genes *AtNGA1* and *AtNGA4* are expressed in the distal parts of the leaves and floral organs, specifically when the style and stigma tissues begin to differentiate [21].

Based on cis-element analysis of the *MtNGA1* promoter, changes in the expression of *MtNGA1* under different abiotic stresses were further detected by qRT-PCR. As an osmotic agent, PEG6000 has a large molecular weight, does not cross the plant cell membrane or cell wall, reduces water absorption, and can simulate stress effects similar to those of soil drought [31,32]. The level of *MtNGA1* transcription was significantly different when exogenous stress treatment (PEG and NaCl) was applied to the *M. truncatula* plant, indicating that MtNGA1 may be involved in regulation of the plant response to stress, providing a basis for stress treatment of transgenic plants. In Arabidopsis, the expression levels of *AtNGA1*–*4* in the roots and shoots of wild-type plants during dehydration stress were altered in different ways and both upregulated and downregulated [25]. There have been few studies on regulation of the NGA family under abiotic stress, which may become a research direction in the future.

Previous studies have shown that *NGA* genes play an essential role in gynoecium development, lateral organ growth and cell proliferation. EcNGA and NbNGA regulate development of the style, stigma and perianth, and the carpel [30]. Four *NGA* genes in Arabidopsis have a general function in the regulation of lateral organ growth, such as the growth of shorter and wider sepals and petals, and wider and more serrated rosette leaves [21,22]. Because BrNGA1 negatively regulates the rate and duration of cell proliferation during organogenesis, Arabidopsis plants overexpressing *BrNGA1* contained smaller and narrower leaves, flowers and cotyledons than wild-type plants and severely retarded root growth [17]. Overexpression of *MtNGA1* in Arabidopsis plants retarded reproductive growth and decreased the number of bolting. The three transgenic Arabidopsis lines bolted 8–10 days later than wild-type Arabidopsis. Then, we detected the expression of flowering related genes: *AtGI*, *AtCO*, *AtFT*, *AtSOC1* and *AtTFL1* in transgenic *MtNGA1* plants under normal conditions. The FLAVIN-BINDING, KELCH REPEAT, F-BOX 1 (FKF1-GI) complex binds the *CO* promoter [33], and then regulates *CO* transcription expression [34]. CO is an immediate activator of FT [35]. SOC1 acts downstream of FT, and the activation of *SOC1* is conducive to the expression of *FT* in the leaves to the meristem, initiating flowering [34]. However, TFL1 has the opposite regulatory effect, and represses flowering [35]. Our results showed that the expression of *AtGI*, *AtCO*, *AtFT* and *AtSOC1* was downregulated, while the expression of *AtTFL1* was upregulated in *MtNGA1* transgenic lines compared to wild-type plants under normal growth conditions. These results demonstrated that MtNGA1 is involved in the long-distance signaling pathway of flowering.

**Figure 10:**
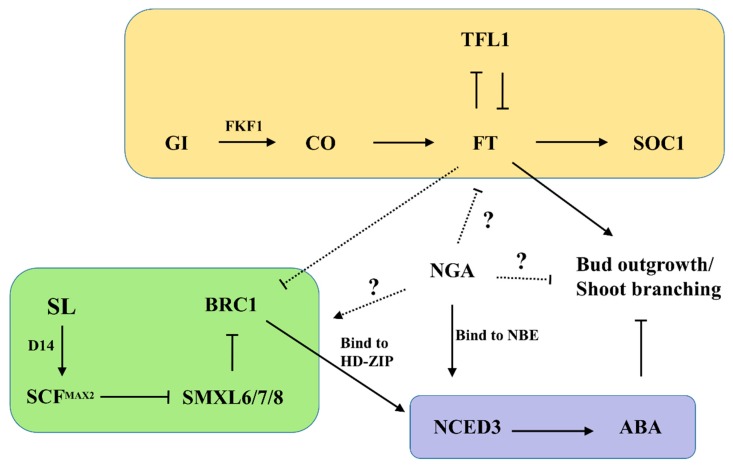
Proposed role of NGA in regulating bud outgrowth/shoot branching in monocotyledons and dicotyledons. GI, GIGANTEA. CO, CONSTANS. FT, Flowering locus T. SOC1, SUPPRESSOR OF OVEREXPRESSION OF CONSTANS 1. TFL1, TERMINAL FLOWER 1. FKF1, FLAVIN-BINDING, KELCH REPEAT, F-BOX 1. SL, Strigolactone. D14, DWARF14. SCF^MAX2^, SCFMAX2 complex. SMXL, SMAX1-LIKE6. MAX, MORE AXILLARY GROWTH. BRC, BRANCHED. NBE, NGA-binding element. NCED3, NINE-CIS-EPOXYCAROTENOID DIOXYGENASE. ABA, abscisic acid. HD-ZIP, Homeodomain-leucine zipper.

Compared to WT plants, the *MtNGA1* transgenic Arabidopsis lines revealed reduced primary branch, secondary branch, tertiary branch and total branch numbers. Therefore, through qRT-PCR assays, we tested variations in the expression of genes that regulated the branch signaling pathway in the *MtNGA1* transgenic plants under normal conditions: *AtSMXL6*, *AtSMXL7*, *AtSMXL8*, *AtMAX1*, *AtMAX2*, *AtBRC1* and *AtBRC2*. Strigolactone (SL) regulates shoot branching through degrading the SMXL6/7/8 protein, in turn reducing inhibition of the transcriptional activation of *BRC1* and *BRC2*, which results in repressed bud outgrowth [36]. Meanwhile, after strigolactone binds DWARF14 (D14), D14 interacts with the Skp1-Cullin-F-box and MORE AXILLARY GROWTH2 (SCF^MAX2^) complex, leading to ubiquitination and degradation of the SMXL6/7/8 proteins via the 26S proteasome [37]. MAX1 and MAX2 affect the repression of axillary shoots through inhibiting primordia formation by the axillary meristem [38]. Our results showed that the expression of *AtSMXL6/7/8* was decreased, while the transcript levels of *AtMAX1/2* and *AtBRC1/2* were increased in *MtNGA1-*overexpressing plants compared to WT plants under normal conditions. These results are in accordance with the identified branch pathway, which suggests that MtNGA1 plays an important role in the branch regulation pathway (Figure 10).

Plants are subject to many environmental stresses during their growth, and some transcription factors, enzymes, and metabolites [39] have been shown to be involved in the plant’s response to adversity. To further study the function of the *MtNGA1* gene, we tested the resistance of *MtNGA1* transgenic Arabidopsis to stress. The resistance of *MtNGA1* transgenic Arabidopsis to mannitol-induced osmotic stress and salt stress was stronger than that of wild-type Arabidopsis. Although mannitol can enter cells and affect metabolism, its short-term use as an osmotic agent can mimic the effects of water stress [32]. Under the osmotic stress induced by mannitol, the leaves of *MtNGA* transgenic plants were dark green and had higher fresh weight, compared with wild Arabidopsis. This phenomenon may be due to the transgenic plants under stress maintaining a higher chlorophyll content, and thus maintaining a higher photosynthetic efficiency. Under water stress conditions, chlorophyll biosynthetic intermediate accumulation in plant leaf cells is reduced, affecting the chlorophyll synthesis pathway, and the accumulation of reactive oxygen species accelerates the degradation of chlorophyll, resulting in a decline in chlorophyll accumulation and blocking of the photosynthetic energy conversion system. This eventually reduces the photosynthetic efficiency, affecting plant growth [40,41,42] (Figure 7B). Furthermore, we examined the expression levels of the genes *AtPLC1*, *AtPLC3*, *AtPLC4*, *AtPLC5*, *AtNCED3* and *AtDI21* in transgenic *MtNGA1* plants under normal conditions. The *AtPLC* genes are induced by various environmental stimuli, including cold, salt and dehydration, and transcriptional activation of the *AtPLC* genes is crucial to the adaptation of plants to environments of stress [43,44]. *NCED3* and *AtDI21* are involved in ABA-dependent regulation of stress-related pathway [45]. NINE-CIS-EPOXYCAROTENOID DIOXYGENASE 3 (NCED3) is an important enzyme in the ABA biosynthetic pathway and ABA accumulation during drought stress [46]. *NCED3* knockout mutants under drought stress conditions exhibited decreased ABA accumulation and drought stress-sensitive phenotypes [47]. In our study, the expression levels of *AtPLC1*, *AtPLC3*, *AtPLC4*, *AtPLC5*, *AtNCED3* and *AtDI21* were upregulated in *MtNGA1* transgenic plants compared to WT plants. These results suggested that MtNGA1 induced the expression of *AtPLC1*, *AtPLC3*, *AtPLC4*, *AtPLC5*, *AtNCED3* and *AtDI21*. A previous study also demonstrated that AtNGA1 activated the *NCED3* gene by binding to the NGA-binding element (NBE) cis-acting sequence in the promoter of the *NCED3* gene during dehydration stress [25]. Therefore, MtNGA1 may be involved in regulation of the stress-response pathway.

As predicted by cis-element analysis of the *MtNGA1* promoter, MtNGA1 may be involved in the ABA signaling pathway. However, *MtNGA1* transgenic plants showed less sensitivity to ABA treatment than WT plants. Studies have reported that stress-responsive genes regulate stress tolerance ability through ABA-dependent and ABA-independent methods [48]. In Arabidopsis, the accumulation of NGA1 proteins in both WT and ABA-deficient mutant plants was increased by dehydration stress, which indicated that NGA1 plays an ABA-independent posttranslational regulatory role in drought stress [25]. Therefore, MtNGA1 may be involved in regulation of the environmental stress response by ABA-independent means.

In summary, MtNGA1 is involved in the regulation of plant growth and development, and abiotic stress. MtNGA1 may have a variety of functions and may have some gene-specific functions. Hence, the function of MtNGA1 needs further study.

## 4. Materials and Methods

### 4.1. Plant Materials and Growth Conditions

*M. truncatula* R108 and wild-type Arabidopsis (Col-0) seeds were provided by the Lawn Research Institute of Beijing Forestry University. The *M. truncatula* seeds were sterilized with 75% alcohol for 10 min, washed with sterile water, germinated on filter paper, and then transferred to Hoagland nutrient solution for subsequent growth under conditions of 16 h light at 26 °C/ 8 h dark at 24 °C. The Arabidopsis seeds were sterilized with 10% NaClO for 10 min, washed with sterile water, and then transferred to 1/2 MS medium (pH 5.8) for germination (transgenic seeds grew on medium supplemented with selected antibiotics). After vernalization for 2 d at 4 °C, the medium was transferred to an incubator and incubated under a 16 h light/8 h dark cycle at 22 °C. After the third and fourth leaves appeared on the seedlings, the seedlings were transferred to sterilized soil or culture medium to continue growth.

### 4.2. Identification and Cloning of NGA1 from M. truncatula

Total RNA was extracted from *M. truncatula* seedlings with a plant RNA isolation kit (Promega, USA), and first-strand cDNA was synthesized with a reverse transcription kit (Takara, Dalian, China). Based on the sequence of NGA1 (MTR_8g023990), two primers (NGA1-F and NGA1-R) were designed to clone the DNA fragment including the complete CDS. The PCR products were ligated into the cloning vector pEASY-NGA1 for storage and further experiments.

### 4.3. Bioinformatics Analysis

A total of 16 NGA protein sequences from *M. truncatula* and other species were obtained, and then the neighbor-joining (NJ) statistical method in MEGA version 6.0 (Koichiro Tamura, Hachioji, Tokyo, Japan) and Bootstrap analysis with 1000 replications were used to construct the phylogenetic tree. The cis-acting elements of the *NGA1* promoter were identified by PlantCARE (http://bioinformatics.psb.ugent.be/webtools/plantcare/html/) and TBtools version 0.6695 (Chen Chengjie, GuangZhou, GuangDong, China).

### 4.4. Subcellular Localization Determination

The completed coding region of *MtNGA1* was amplified by PCR with the SAT-NGA1-F and SAT-NGA1-R primers (Supplementary Table S1). The product was ligated to pSAT6-GFP with a seamless ligase, and the pSAT6-35S::*MtNGA1*-*GFP* vector was constructed. The recombinant plasmid was transformed into the mesophyll protoplasts of *M. truncatula*. The preparation and transformation of mesophyll protoplasts were carried out as described previously [26,49]. After 18 h of incubation in the dark, the transformed protoplasts were detected by a confocal laser scanning microscope.

### 4.5. Abiotic Stress and ABA Treatment

For different treatments, whole seedlings of 20-day-old *M. truncatula* grown in Hoagland nutrient solution were moved to new Hoagland nutrient solution with a final concentration of 200 mM NaCl, 15% PEG, or 100 μM abscisic acid (ABA). Samples were collected at 0 h, 2 h, 4 h, 8 h, 12 h and 24 h, and total RNAs was isolated for further experiments.

### 4.6. Quantitative Analysis

First-strand cDNA was used as a template for real-time quantitative RT-PCR (qRT-PCR) analysis. Actin, a housekeeping gene in *M. truncatula*, was used as the internal reference, and the relative expression of *NGA1* was determined by the 2^-ΔΔCT^ method [50]. Three biological repeats were performed. See Supplementary Table S1 for primer sequences.

### 4.7. Obtaining Transgenic Plants

A fragment containing the complete CDS was amplified by PCR with the primers 3301-NGA1-F and 3301-NGA1-R (Table S1), and then ligated into the pCAMBIA3301 (p3301) vector, generating the plant overexpression vector p3301-35S::*NGA1*. The constructed vector was transformed into Arabidopsis through the floral dip method [51]. T3 transgenic lines with high expression levels of *MtNGA1* were generated by self-pollination for subsequent experiments.

### 4.8. Stress Analysis of Transgenic Plants

Three different transgenic lines with high expression levels of *NGA1* were selected. Transgenic Arabidopsis seedlings were cultured on 1/2 MS medium for 5 days under normal condition with a 16 h light/8 h dark cycle, and then transferred to 1/2 MS medium containing 500 mM mannitol, 200 mM NaCl or 20 μM ABA. After 7 days of mannitol treatment, the root lengths and fresh weights of all lines were measured. Seedlings treated with NaCl were assessed 3 days after, and surviving seedlings were counted. The ABA-treated lines were observed and assessed 12 days later. Furthermore, changes in the expression of *AtPLC*, *AtDI21* and *NCED3* were detected by qRT-PCR.

## 5. Conclusions

In summary, through bioinformatic analysis of the *M. truncatula* genome, we identified, cloned, and transformed the *MtNGA1* gene from *M. truncatula* into Arabidopsis by constructing an expression vector. Expression analysis showed that under normal growth conditions, the expression level of *MtNGA1* in each organ of wild-type *M. truncatula* plants were different and significantly affected by exogenous stress. Exogenous PEG and NaCl treatment significantly increased the expression level of *MtNGA1*. Analysis of transgenic plants showed, for the first time, that overexpression of *MtNGA1* reduced branching and delayed flowering in Arabidopsis. Transgenic plants showed strong tolerance to exogenous stress (mannitol, NaCl) and low sensitivity to ABA. Therefore, we infer that MtNGA1 affects the accumulation of NCED3 by binding NBE elements, as well as plant branching and flowering by other means. Analysis of these specific mechanisms needs to be supported by follow-up research. This study lays a foundation for in-depth functional research and application of the *MtNGA1* gene.

## Supplementary Materials

Supplementary materials can be found at https://www.mdpi.com/1422-0067/21/7/2384/s1.
