# Supplementary material for: Expression of a NGATHA1 Gene from Medicago truncatula Delays Flowering Time and Enhances Stress Tolerance
Source: Int J Mol Sci. 2020 Mar 30;21(7):2384. doi: 10.3390/ijms21072384 (PMC7177866; doi:10.3390/ijms21072384)
Supplement: Supplementary file 1 [file ijms-21-02384-s001.pdf]

Supplementary Material

Table S1. Primer sequences used for gene cloning, plasmid construction and qRT-PCR.

Primer name	Primer sequence(5′-3′)	
	F	R
**For gene clone**		
MtNGA1	GTCAAATATGAAGGCACCGACTC	ATCAACCCAACAAGGCTAACAGA
**For GFP fusion**		
SAT-NGA1	CACCATTTACGAACGATAGCATGGAGTTGATGCAAGAAGT	CTAGTCAGATCTACCATCCCTTGTTGTTGGTAATTATGAT
**For overexpression**		
3301-NGA1	GAACACGGGGGACTCTTGACATGGAGTTGATGCAAGAAGTGA	TTTACCCTCAGATCTACCATTTGTTGTTGGTAATTATGATAT
**For qRT-PCR**		
qMtNGA1	GATGCTGGCGACATTGTTTC	GGAGTGAAAAGTGTTGCTGATG
qAtNCED3	AGAACAAGGTCGCAAGATTCGGGATT	GAGATTCAGGCGGATTTCAGACAGGA
qAtDI21	TGTAACAGCAGCAGGATTGAGTAAGG	CGCACGATTGGAAGGTCTGTAGTATC
qAtPLC1	CGGTTAAGCATGAGTGAGCA	ATGAATCCACCCAACGAGAG
qAtPLC3	CAAGGACATGGGAAGCAACT	CTTTTGCAAGGGTCGAAGAG
qAtPLC4	AACTTGCTCTGCTCCGTGTT	AAGAGTGGAACAGCGCGTAT
qAtPLC5	CAAAAGACATGGGAGCCATT	ACCCGAGAAATCGTCCTTCT
qAtSMXL6	GATTGCCAAGGATAATGCCACTGACC	GCCCGATGATAATGATGACCATGAAGC
qAtSMXL7	ACATTAAACTCGACGTGCTTCATCCT	AACCATCCCTGACTTCAATCTCCCTA
qAtSMXL8	GGACCTGGACTACTCTTACACTACGG	CACATTCGGAAACCTTCTCATCATCT
qAtMAX1	CAAGGGAAACTGCTAAAGAAGTGGAG	CCGAATGGGATGAAAGCGTATGGATG
qAtMAX2	TTTTGAACACTGTGGTGGTTTCCTTG	ACGATTTCGCTTGATTTGTATCCCTC
qAtBRC1	CCACTGAGCCCTCGGAAACTATAAAC	GTCTCATCCTACGATCTCGTGTCCCT
qAtBRC2	TTCTCACCATCACTACTCTTCGCCTTCT	TTTGACCGTCCTTCTCCATTGAACCT

qAtGI	TGGGTTGATGAGATGTAAGTGGGATA	AACAGTGAAACAACGGAGAAAGACAG
qAtCO	ACTCTTTCAGCTCCATGACCACTACTC	TTCAAGTTTAAGCGGAACAACTCTATC
qAtFT	ACCCTCACCTCCGAGAATATCTCCAT	CTAAAGTCTTCTTCCTCCGCAGCCAC
qAtSOC1	AGGCATACTAAGGATCGAGTCAGCAC	GAAGAACAAGGTAACCCAATGAACAA
qAtTFL1	GAACACCTGCACTGGATCGTTACAAA	CGACAGGGAGACCAAGATCATACTCG
qAtActin	ACCACTGTCCACTCTATCACTGC	TGAGGGATGGCAACACTTTCCC
qMtActin	TGGGCTGCCACAGAACATTTGA	GCTGTGGTTGCTTTTTTGGTGTCTC
